# SARS-CoV-2–specific mucosal immune response in vaccinated versus infected children

**DOI:** 10.3389/fcimb.2024.1231697

**Published:** 2024-03-27

**Authors:** Maria Giulia Conti, Eva Piano Mortari, Raffaella Nenna, Alessandra Pierangeli, Leonardo Sorrentino, Federica Frasca, Laura Petrarca, Enrica Mancino, Greta Di Mattia, Luigi Matera, Matteo Fracella, Christian Albano, Carolina Scagnolari, Martina Capponi, Bianca Cinicola, Rita Carsetti, Fabio Midulla

**Affiliations:** ^1^ Department of Maternal, Child Health and Urological Sciences, Sapienza University of Rome, Rome, Italy; ^2^ B Cell Unit, Immunology Research Area, Bambino Gesù Children’s Hospital, Istituti di Ricovero e Cura a Carattere Scientifico (IRCCS), Rome, Italy; ^3^ Laboratory of Virology, Department of Molecular Medicine, Sapienza University of Rome, Rome, Italy

**Keywords:** SARS-CoV-2, COVID-19 vaccines, children, immunogenicity, mucosal immunity

## Abstract

The anti-COVID-19 intramuscular vaccination induces a strong systemic but a weak mucosal immune response in adults. Little is known about the mucosal immune response in children infected or vaccinated against SARS-CoV-2. We found that 28% of children had detectable salivary IgA against SARS-CoV-2 even before vaccination, suggesting that, in children, SARS-CoV-2 infection may be undiagnosed. After vaccination, only receptor-binding domain (RBD)–specific IgA1 significantly increased in the saliva. Conversely, infected children had significantly higher salivary RBD-IgA2 compared to IgA1, indicating that infection more than vaccination induces a specific mucosal immune response in children. Future efforts should focus on development of vaccine technologies that also activate mucosal immunity.

## 1 Introduction

Children’s mucosal immunity is highly dynamic and starts developing early in life, when the respiratory and gastro-intestinal tracts are exposed to the multitude of microorganisms that the infant is going to face for the first time (Brandtzaeg, 2013). The rapid reactivity of mucosal responses is already observed in infants, when Spike-specific IgA can be detected in the saliva of children born to SARS-Cov-2–infected mothers (Conti et al., 2021).

While COVID-19 may be a severe and lethal disease in adults, the likelihood of serious disease in children is much lower than in adults, even though it can be a severe infection also in children (Mehta et al., 2020). A strong innate immune system, natural antibodies, cross-reactive immunity, and preparedness to the encounter with unknown pathogens are the instruments of protection necessary for the survival of the infants, for whom all microorganisms are novel (Levy, 2007).

IgA mainly exists at mucosal surfaces, providing the first line of immune defense against pathogens invasion. Moreover, IgA is also an important serum immunoglobulin, second only to IgG, which can mediate anti- or pro-inflammatory response (Yoo and Morrison, 2005). It has been shown from previous studies that IgA has a strong antiviral effect, much greater than that of IgG, which may be related to IgA high level of sialylation on the glycochains (Steffen et al., 2020). Anti-SARS-CoV-2–specific salivary IgA is measurable for months in adults that were hospitalized for COVID-19 (Isho et al., 2020). The importance of salivary IgA has been also suggested by the study of breakthrough infections in individuals fully immunized against SARS-CoV-2 (Terreri et al., 2022). Salivary Spike-specific IgA rapidly increases in response to natural infection but is very low in the saliva of adult vaccinated individuals. This finding may explain why vaccination protects against severe disease but is less effective in preventing contagion (Bergwerk et al., 2021). Human IgA is produced in two subtypes, which differ in their glycosylation sites, IgA1 and IgA2: both IgA1 and IgA2 monomers contain multiple N-glycosylation sites, and IgA1 has another nine potential O-glycosylation sites in the hinge region. IgA1 are more abundant in the serum (80%), while IgA2 are relatively higher in the saliva and colostrum (Steffen et al., 2020). IgA2 is more stable than IgA1 in the mucosal system due to the lack of a highly O-glycosylated hinge region that is susceptible to the protease in bacteria (Senior and Woof, 2005). IgA1 and IgA2 are normally found in secreted fluids in a dimeric form (Cerutti and Rescigno, 2008). Secretory IgA is produced as a dimer by mucosal plasma cells and is transported through the epithelia by the polymeric Ig receptor (Brandtzaeg, 2007). The anti-COVID-19 BNT162b2 vaccine induces a strong systemic response so that antibodies from the serum can transudate to the saliva (Sheikh-Mohamed et al., 2022). Despite effective protection against COVID-19, in adults, parenteral vaccination is not able to induce a significant anti-SARS-CoV-2 mucosal immune response, as documented by lower levels of IgA2 in the saliva of vaccinated compared to COVID-19 adult patients (Darwich et al., 2022).

Nonetheless, mRNA vaccination efficiently induced secretory IgA in adults previously infected with SARS-CoV-2 (Sano et al., 2022). We have recently demonstrated that vaccination induced a potent humoral and cellular immune response in all children 5–11 years old who received the BNT162B2 vaccine (Cinicola et al., 2022); however, children’s mucosal immune response to the Spike protein after vaccination or natural infection has not been characterized yet.

In this study, we investigated children’s mucosal immune response to SARS-CoV-2 vaccination compared to natural infection by measuring Spike-specific IgA in the saliva of both vaccinated and infected children. We further dissected IgA immune response in children by detecting receptor-binding domain (RBD)–specific IgA1 and RBD-specific IgA2. Finally, we compared systemic (serum) and mucosal (salivary) antibody levels in a subgroup of infected versus vaccinated children.

## 2 Methods

### 2.1 Study populations

We designed a prospective study enrolling children who received the BNT162b2, Pfizer/BioNTech vaccine at the pediatric vaccine center of Policlinico Umberto I hospital (range: 5–11 years), [vaccinated cohort (VAX-C)]. We retrospectively analyzed data of serum and saliva Spike-specific antibodies of a cohort of age-matched children previously enrolled at Policlinico Umberto I hospital who tested positive for SARS-CoV-2, [infected cohort (INF-C)]. The study was approved by the Policlinico Umberto I Ethic Committee (reference number 5839, protocol number 0621/2020).

### 2.2 Samples collection

Serum and saliva samples of the INF-C cohort were collected 20 days after the first positive SARS-CoV-2 polymerase chain reaction (PCR) molecular test. Saliva samples of the VAX-C cohort were collected on the day of the first vaccine dose (T0) and 10 days after the second vaccine dose (T1) by a saliva collection device (Self LolliSponge, Copan Italia S.p.A.). Peripheral blood samples were collected by venipuncture at T1. Samples were stored at −80°C until further analysis.

### 2.3 Clinical data collection

A structured questionnaire was submitted to the parents of the children to register the following information: date of birth, previous SARS-CoV-2 infection, close contact to a SARS-CoV-2–positive family member, symptoms suggestive of an upper respiratory tract infection over the 10 days before T1.

### 2.4 Measurement of Spike-specific IgA and IgG

A semiquantitative determination of human IgA antibodies, in both saliva and serum samples, and IgG in the saliva samples, against SARS-CoV-2 was performed at a dilution of 1:20 (saliva) and 1:100 (serum), adapting the Anti-SARS-CoV-2 Spike ELISA (EUROIMMUN), according to the manufacturer’s instructions. Samples were diluted in sample buffer and dispensed in Spike-coated wells for 1h at 37°C. Positive control, negative control, and a calibrator provided by the kit were added. Wells were washed three times using a washing buffer (PBS+ 0.05% Tween 20) and horseradish peroxidase (HRP)–conjugated anti-human IgG or IgA were added and incubated for 30 min at 37°C. Last, wells were washed 3 times with washing buffer and HRP substrate was added for 15 min at room temperature. Stop solution was added. Values were then normalized for comparison with a calibrator. Results were evaluated by calculating the ratio between the extinction of samples and the extinction of the calibrator. Negative controls were children enrolled in Policlinico Umberto I hospital who tested negative for SARS-CoV-2 RNA in nasopharyngeal swabs. Moreover, serum from negative controls were tested for anti-S IgG and IgA to exclude previous SARS-CoV-2 infections. By testing saliva of negative controls, we established the cutoff value for positive salivary samples, calculated as the mean values of 16 negative saliva OD ratios plus 2 standard deviations; serum cutoff was established as manufacturer’s instructions. A quantitative determination of serum IgG against SARS-CoV-2 was performed at a dilution of 1:200 using Quantitative Anti-SARS-CoV-2 Spike ELISA (EUROIMMUN, Lübeck, Germany).

### 2.5 Measurement of RBD-specific IgA1 and IgA2

IgA1 and IgA2 antibodies against SARS-CoV-2 RBD were evaluated in saliva samples using an in-house ELISA. Ninety-six well plates (Corning, Corning, NY, USA) were coated overnight with 1 μg/ml of purified SARS-CoV-2 RBD protein (Sino Biological, Beijing, China). After the washing step (PBS + 0.05% Tween 20), we performed a blocking of 1h at 37°C with PBS+1% BSA+ 0.05% Tween 20. Following a washing step, plates were incubated for 2h at 37°C with six threefold serial dilutions (starting from undiluted) of saliva samples. After washing, plates were incubated for 1h at 37°C with HRP-conjugated mouse anti-human IgA1 or IgA2 antibody (Bio-Rad, Hercules, CA, USA). The assay was developed with o-phenylen-diamine tablets (Sigma-Aldrich, St. Louis, MO, USA) as a chromogen substrate. Optical density (OD) was measured using a microtiter plate reader at a wavelength of 450 nm. Since at the time of the study, anti-RBD human IgA1 and IgA2 were unavailable; we were not able to quantify the level (not the exact concentration) of anti-RBD IgA1 and IgA2 and, instead, we compared the area under the curve for each sample.

### 2.6 Statistical analysis

Data were analyzed and represented in dot plots [median with Interquartile range (IQR)] using GraphPad Prism 9 version 9.3.1. Demographics were summarized with descriptive statistics [median(min–max) or percentage (%) for continuous values]. Immunological data were compared between the different study times and study groups. Study time and groups were compared by the Wilcoxon matched pair signed-rank test or the two-tailed Mann–Whitney U-test were used. Differences were deemed significant when *p* < 0.05.

## 3 Results

We enrolled 100 children (5–11 years) consecutively admitted at the pediatric vaccine center of Policlinico Umberto I hospital from February to April 2022 to receive the first dose of the BNT162b2, Pfizer/BioNTech vaccine. We collected saliva samples (*n* = 90) right before the first vaccine dose was administered (T0). 91 children came back for the second vaccine dose, 21 days after the first dose. About 10 days after the second dose (T1), 60 children returned to follow up, and we collected 58 saliva samples and 22 serum samples [median days from first vaccine dose to T1 was 34 (30–51); median days from second vaccine dose to T1 was 11 (9–30)]. We also collected saliva and serum samples from 41 children (5–13 years) who tested positive by PCR to SARS-CoV-2 between October 2020 and March 2021 (median days from the first PCR positive test to T1 was 21 (range: 8–42). Clinical and demographic characteristics of the study participants are described in **Tables 1** and **2**.

**Table 1:** Characteristics of the vaccinated cohort.

Vaccinated children VAX-C no. = 100		
	T0 no. = 90	T1 no. = 58
Age, median (min–max), years	8 (5–11)	8 (5–11)
Male, no. (%)	52 (57, 7)	30 (50, 8)
Days from first vaccine dose (T0) to T1, median (min–max)	na	34 (30–51)
Days from second vaccine dose to T1, median (min–max)	na	11 (9–30)
Paired T0–T1 samples	na	49
SARS-CoV-2 infected before vaccination, no. (%)	8 (8, 8)	8 (13, 5)
SARS-CoV2 infection severity		
Asymptomatic, no.	5	5
Symptomatic, no.	3	3
Days from infection to T0, median (min–max)	463,5 (224–494) (15 months)	na
SARS-CoV-2 exposure before vaccination, no. (%)	15 (16, 6)	17 (28, 8)
Days from exposure to T0, median (min–max)	60 (30–425)	

T0 = day of the first saliva collection; it corresponds to the day of the first vaccine dose; T1= day of the second saliva collection, approximately 10 days after the second vaccine dose; na, not applicable.

**Table 2:** Characteristics of the infected cohort.

Infected children INF-C no. = 41	
Age, median (min–max), years	10 (5–13)
Male, no. (%)	26 (63, 4)
SARS-CoV2 infection severity	
Asymptomatic, no. (%)	20 (48, 7)
Symptomatic, no. (%)	21(51, 3)
Days from infection to T0, median (min–max)	21 (8–42)

T0 = day of saliva collection after positivity to SARS-CoV-2 PCR from nasopharyngeal swab sample.

### 3.1 Salivary SARS-CoV-2 specific antibodies in vaccinated children

**Figure 1:**
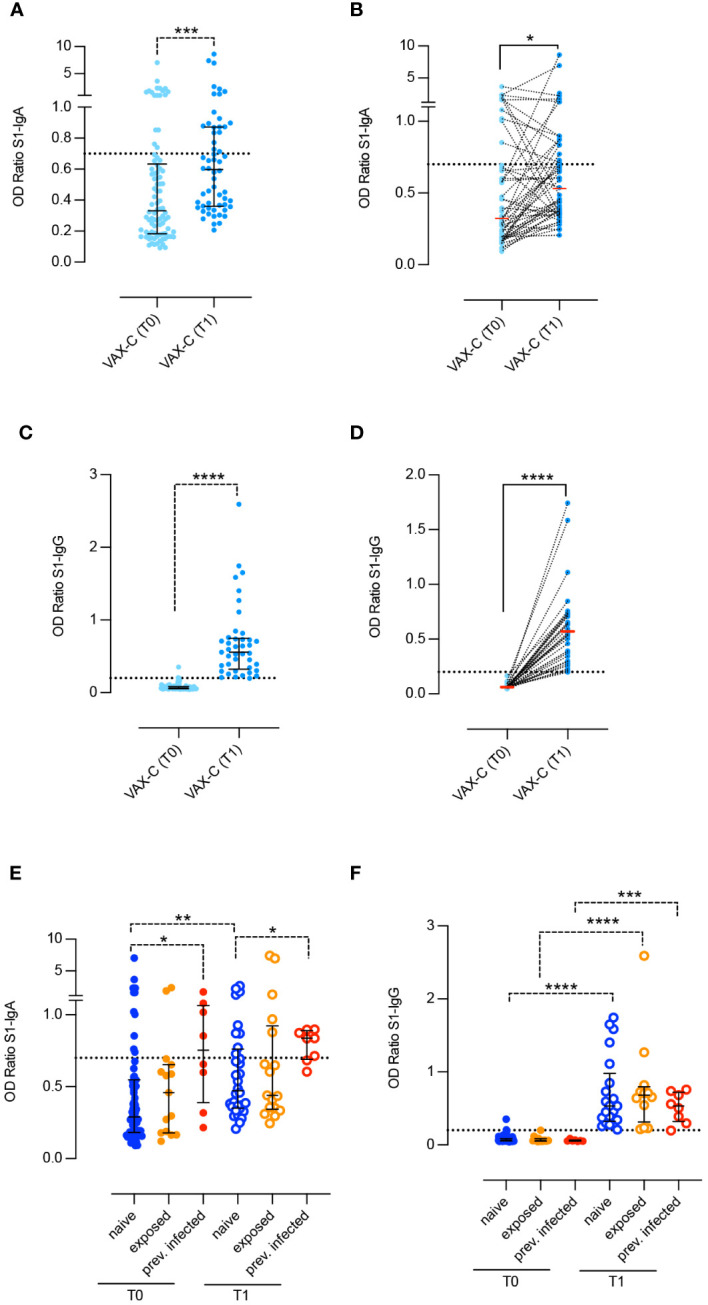
Spike-specific antibodies in the saliva of children before and after two doses BNT162b2, Pfizer/BioNTech vaccine. Anti-S1–specific IgA and IgG **(A–D)** were measured in the saliva of children right before the administration of the first dose (VAX-T0, *n* = 90) and 10 days after the second dose (VAX-T1, *n* = 59) of the BNT162b2, Pfizer/BioNTech vaccine. For 49 children, we were able to obtain paired T0–T1 samples **(B, D)**. **(E, F)** VAX-C was stratified based on the history of a previous SARS-CoV-2 infection (previously infected, red dots; *n* = 8), close contact with a positive family member (exposed, orange dots; *n* = 15 at T0 and *n* = 17 at T1) or none of the above (naïve, blue dots; *n* = 67 at T0 and *n* = 34 at T1). Cutoff (dotted line) was calculated as the mean plus 2 standard deviations in 16 negative control samples. Medians **(A–F)** and IQR **(A, C, E, F)** are indicated, and statistical significance was determined using Wilcoxon matched-pairs signed rank test (continuous line) or with the unpaired Mann–Whitney U test (dotted line). *p < 0.05, **p < 0.01, ***p < 0.001, ****p < 0.0001.

Spike specific salivary IgA significantly increased after vaccination (*p* = 0.0001) (**Figure 1A**). The increase is also significant (*p* = 0.0316) when analyzing paired saliva samples, although the increase cannot be appreciated for all subjects, as shown in **Figure 1B**. On the other hand, Spike-specific salivary IgG were not detectable for all subjects at T0 and significantly increased after the second dose (*p* < 0.0001) (**Figure 1C**). The increase remained significant (*p* < 0.001) when we analyzed paired samples (**Figure 1D**). IgA and IgG levels in the saliva were not significantly correlated (data not shown, *R* = 0.056 *p* = 0.07).

At T0, 28% of children already had either measurable (20%) or borderline (8%) values of Spike specific IgA in the saliva. At T1, the frequency of IgA positive samples increased to 35.5% and that of children with borderline levels to 13.5%.

We analyzed the levels of Spike-specific salivary IgA and IgG at T0 and T1 after stratifying the results according to the children history of SARS-CoV-2 (**Figures 1E, F**). Naïve children (naive-VAX) had no history of previous infection or contact with infected individuals (*n* = 67; 74, 6%), fifteen (16.6%) were considered exposed to SARS-CoV-2 (exposed-VAX), and eight children had experienced COVID-19 (8.8%), with an either asymptomatic or mild clinical course, several months before vaccination (infected-VAX). Spike specific IgA significantly increased in response to vaccination in the naïve-VAX (*p* = 0.0019). In the exposed-VAX and infected-VAX groups, the increase was not significant (**Figure 1E**). In contrast, children previously infected with SARS-CoV-2 had significantly higher Spike-specific salivary IgA before (*p* = 0.018) and after (*p* = 0.02) vaccination compared to naïve and exposed children (**Figure 1E**).

Spike-specific salivary IgG was not detectable before vaccination in any of the three groups, thus suggesting that previous SARS-CoV-2 infection or exposure to an infected relative does not influence the concentration of specific IgG in the saliva. After vaccination, the increase of IgG is significant in all groups (naïve-VAX and exposed-VAX *p* < 0.001; infected-VAX *p* = 0.0003) (**Figure 1F**).

### 3.2 Immune response in vaccinated versus naturally infected children

**Figure 2:**
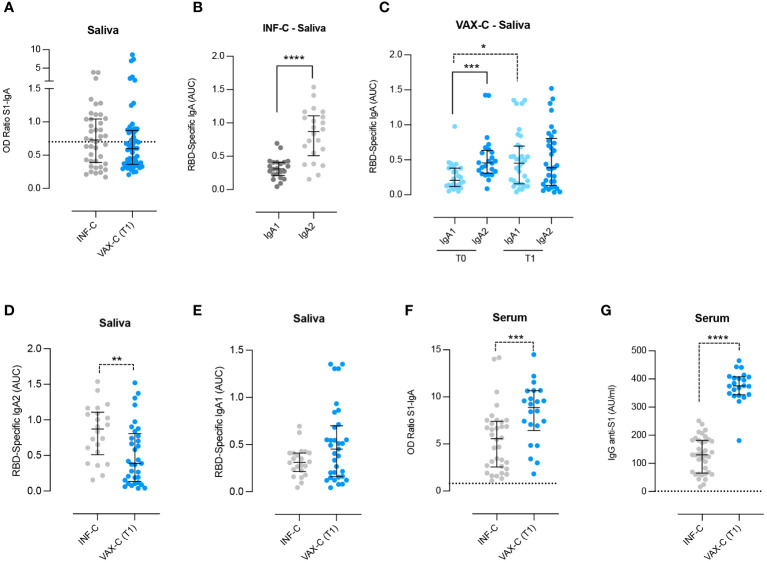
Detection of anti-RBD IgA1 and IgA2 in the saliva and Spike-specific antibodies in the serum and saliva of infected and vaccinated children. **(A)** Anti-Spike–specific IgA in the saliva of infected (INF-C; *n* = 41, saliva samples collected a median of 21 days after first SARS-CoV-2–positive nasopharyngeal swab sample) versus vaccinated children at T1 [VAX-C (T1); *n* = 58, saliva samples collected a median of 11 days after the second vaccine dose]. **(B)** Anti-RBD specific IgA1 and IgA2 in the saliva of infected children who were not previously vaccinated and that had anti-Spike IgA in the saliva above the cutoff value (*n* = 22). **(C)** Anti-RBD-specific IgA1 and IgA2 in the saliva of vaccinated children (VAX-C), before and after vaccination with the BNT162b2, Pfizer/BioNTech vaccine (T0 and T1, respectively). For both times, we selected only the saliva samples in which Spike-specific IgA were detectable T0 *n* = 24 and T1 *n* = 33. **(D, E)** Anti-RBD–specific IgA2 and IgA1 in the saliva of infected (*n* = 22) versus vaccinated children at T1 (*n* = 33). **(F, G)** Spike-specific IgA and IgG were measured in the serum of INF-C (*n* = 34) and VAX-C at T1 (*n* = 22). Cutoff (dotted line) was calculated as the mean plus 2 standard deviations in 16 negative control samples. **(A–G)** Medians and IQR are indicated, and statistical significance was determined using the Wilcoxon matched-pairs signed rank test (continuous line) or with the unpaired Mann–Whitney U (dotted line) test. *p < 0.05, ***p < 0.001, ****p < 0.0001. **(D)** 0.07 is significant considering a confidence level of 93% instead of 95%. **p < 0.01

We also collected the saliva of children who tested positive for COVID-19 between October 2020 and March 2021 before children vaccination was implemented (INF-C). We aimed to compare the ability to generate Spike-specific IgA in the saliva of children with natural infection (INF-C) or parenteral vaccination (VAX-C T1). We found that the level of salivary anti-Spike specific IgA did not differ between the two groups (**Figure 2A**).

Human IgA is produced in two subtypes IgA1 and IgA2. Their distribution differs between serum and mucosal sites, and it has been shown that in COVID-19-infected adults, the levels of specific IgA2 in the saliva are higher compared to those of individuals who were only vaccinated (Darwich et al., 2022). Thus, we measured the concentration of RBD-specific IgA1 and IgA2 in our two cohorts, INF-C and VAX-C. In the VAX-C group, we measured the levels of the two subtypes of IgA only in the subjects that had detectable values of Spike-specific IgA before vaccination.

We found that IgA2 was significantly more abundant than IgA1 in the saliva of INF-C (*p* < 0.0001; **Figure 2B**), thus confirming that IgA2 is increased in response to SARS-CoV-2 infection.

In VAX-C children before vaccination (T0), we found that the levels of specific IgA2 were significantly higher compared to specific IgA1 (*p* = 0.0005) (**Figure 2C**), suggesting that the detectable values of anti-S1 IgA could be due to a previous infection.

Vaccination led to a significant increase of RBD-specific IgA1 (*p* = 0.012) but not IgA2 (*p* = 0.5054) (**Figure 2C**). This result indicates that parenteral vaccination does not influence the levels of salivary IgA2. The increase of IgA1 was confirmed (*p* = 0.07), when we included in the analysis only naive children that had detectable values of anti-S IgA before vaccination (**Supplementary Figure S1**).

As for the adults, the levels of RBD-specific IgA2 were significantly higher in the saliva of naturally infected children compared to fully vaccinated ones (*p* = 0.0054) (**Figure 2D**), while there was no statistically significant difference for RBD-specific IgA1 between the two groups (**Figure 2E**). We also collected serum samples from a subgroup of 21 vaccinated children at T1. Spike-specific IgA levels in saliva and serum were not correlated (ρ = 0.01, *p* = 0.6), data not shown. We also compared serum anti-Spike IgA levels in 21 vaccinated children and 20 age-matched naturally infected children, and found that serum Spike-specific IgA and IgG levels were significantly higher in vaccinated children compared to naturally infected children (*p* = 0.008 and *p* < 0.0001 respectively; **Figures 2F, G**), suggesting that vaccination has a higher capacity to generate serum antibody also of IgA isotype.

## 4 Discussion

Our study suggests that mucosal and systemic responses to SARS-CoV-2 natural infection and vaccination are slightly distinct in children.

Following vaccination, the increase of salivary specific antibodies, both IgA and IgG, is significant. Different from Spike-specific IgG, which were not detectable in the saliva before vaccination but only after, we found that a consistent percentage of children, never infected or exposed to SARS-CoV-2, already had detectable Spike-specific IgA in the saliva even before vaccination. This finding suggests that, in several children, SARS-CoV-2 infection may escape diagnosis, as they usually develop a mild or asymptomatic disease. The presence of specific IgG in the saliva, after vaccination, can be explained by the transudation of vaccine-produced IgG from the plasma to the oral cavity after vaccination (Darwich et al., 2022). Salivary IgA, differently, can either transudate from the plasma to oral secretions or be produced by local plasma cells, induced by an infection. Indeed, children who were vaccinated but also reported a history of a proven previous infection by SARS-CoV-2 had significantly higher Spike-specific salivary IgA compared to vaccinated naïve children suggesting that previous infection may act like a booster for mucosal-specific plasma cells.

We have shown that, in SARS-CoV-2–infected children, there is a specific increase in IgA2-isotype antibodies in the saliva. On the other hand, in children who received the mRNA anti-COVID-19 BNT162b2 parenteral vaccine, there is a slight increase in salivary Spike-specific IgA1, suggesting a different immune response to the vaccine compared to natural infection. These findings highlight the importance of understanding the nuances of the immune response to both infection and vaccination, in order to develop vaccines able to better control and prevent the spread of infectious diseases. Moreover, this result is consistent with the notion that IgA2 represents an important component in natural mucosal immune response. IgA subtypes have been discussed to have different functional effects: in the serum, IgA2 exerts inflammatory effects inducing cytokines production and promoting neutrophils and macrophages inflammatory activity, while IgA1 is important for immune homeostasis (Steffen et al., 2020). In adults, respiratory infections induce the production of specific IgA at the site of pathogen entry, such as the mucosal surface (Sano et al., 2022), vaccination also leads to the presence of specific IgA in the saliva, mainly IgA1 rather than IgA2 (Cinicola et al., 2022).

Although intramuscular vaccination in children does not induce specific IgA2 in the saliva, serum Spike-specific IgA and IgG levels were significantly higher in vaccinated children compared to naturally infected children, confirming the efficacy of vaccination for systemic protection against COVID-19.

Our study has some limitations. First, we reported a high rate of dropout at T1 for the vaccinated cohort. Some of the children enrolled at T0 experienced COVID-19 before the second dose was administered and did not come back. Another explanation for follow-up losses was the parents’ reluctance to return to the hospital during the pandemic.

Second, serum samples were collected only in a subgroup of the vaccinated enrolled children, since the venipuncture was proposed by medical researchers to the parents in addition to the saliva collection but was not required to be involved in the study.

Our results confirm that the parenteral mRNA anti-COVID-19 BNT162b2 vaccine is able to generate a strong systemic immune response as documented by high levels of specific IgA and IgG in the serum of vaccinated children; on the other hand, SARS-CoV-2 infection is associated to the presence of RBD-specific IgA2 in the saliva. The persistently low levels of salivary RBD-specific IgA2 after complete vaccination, suggest that the mRNA vaccines do not boost the IgA2-specific mucosal response in children, similar to what has been found in adults (Darwich et al., 2022). RBD-specific IgA1 instead increases in the saliva of vaccinated children probably as a result of passive leakage from the blood circulation mainly via gingival crevicular epithelium (Brandtzaeg, 2007).

Future efforts should focus on the development of vaccine technologies that robustly activate mucosal immunity in children.

## Data availability statement

The raw data supporting the conclusions of this article will be made available by the authors, without undue reservation.

## Ethics statement

The studies involving humans were approved by Policlinico Umberto I Ethic committee (Reference number 5839, Protocol number 0621/2020). The studies were conducted in accordance with the local legislation and institutional requirements. Written informed consent for participation in this study was provided by the participants’ legal guardians/next of kin.

## Author contributions

MGC conceived the project, designed the study, acquired clinical data, analyzed data, and wrote the manuscript. EPM provided substantial contributions to the conception of the work, performed experiments, analyzed data, and contributed to the writing of the manuscript. RN provided substantial contributions to the conception of the work, provided expertise and reviewed, commented on the manuscript. LS, FF and CA performed experiments, analyzed data, and contributed to the writing of the manuscript. CA: Writing – review & editing. LP, EM, MF, GM, LM, MC, and BC acquired clinical data, and commented on the manuscript. AP and CS provided expertise and reviewed, commented on the manuscript. RC and FM conceived the project, provided expertise, and reviewed, commented on the manuscript. All authors contributed to refinement of the study protocol. All authors contributed to the article and approved the submitted version.

## Supplementary material

Supplementary Figure 1 Anti-RBD–specific IgA1 and IgA2 in the saliva of children supposed to be naive to SARS-CoV-2 but with Spike-specific IgA detectable at T0.
