# Supplementary figures and images for: SARS-CoV-2–specific mucosal immune response in vaccinated versus infected children
Source: Front Cell Infect Microbiol. 2024 Mar 27;14:1231697. doi: 10.3389/fcimb.2024.1231697 (PMC11004290; doi:10.3389/fcimb.2024.1231697)

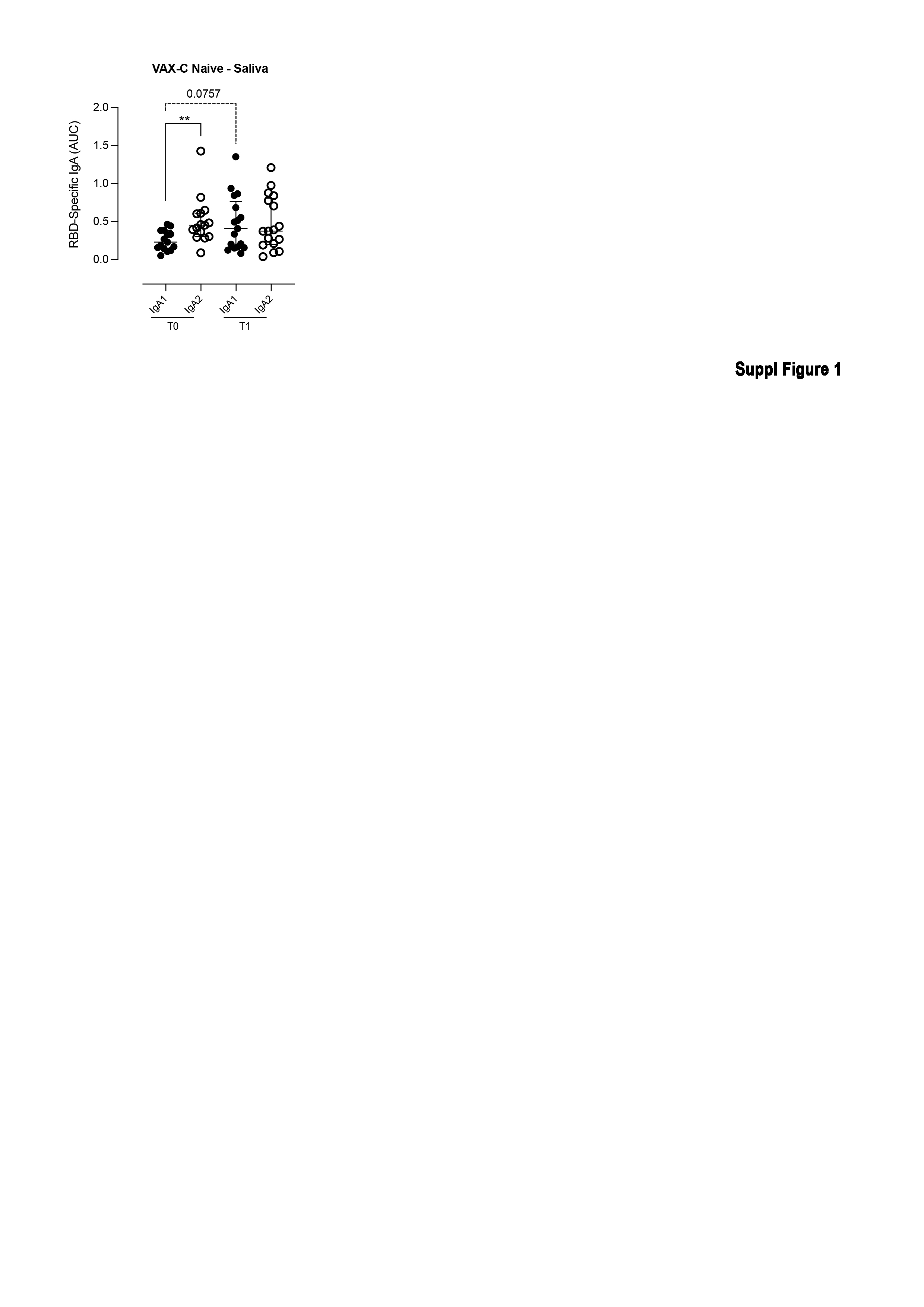

Supplement: Supplementary Figure 1 — Anti-RBD–specific IgA1 and IgA2 in the saliva of children supposed to be naive to SARS-CoV-2 but with Spike-specific IgA detectable at T0. [file Image_1.jpeg]
